# Diverse effects of a low dose supplement of lipidated curcumin in healthy middle aged people

**DOI:** 10.1186/1475-2891-11-79

**Published:** 2012-09-26

**Authors:** Robert A DiSilvestro, Elizabeth Joseph, Shi Zhao, Joshua Bomser

**Affiliations:** 1 Department of Human Nutrition, The Ohio State University, 345 Campbell Hall, 1787 Neil Ave, Columbus, OH, 43210-1295, USA

**Keywords:** Curcumin, Catalase, Nitric oxide, Antioxidant capacity, Antioxidant activity

## Abstract

**Background:**

Curcumin extracts of turmeric are proposed to produce health benefits. To date, human intervention studies have focused mainly on people with existing health problems given high doses of poorly absorbed curcumin. The purpose of the current study was to check whether in healthy people, a low dose of a lipidated curcumin extract could alter wellness-related measures.

**Methods:**

The present study was conducted in healthy middle aged people (40–60 years old) with a low dose of curcumin (80 mg/day) in a lipidated form expected to have good absorption. Subjects were given either curcumin (N = 19) or placebo (N = 19) for 4 wk. Blood and saliva samples were taken before and after the 4 weeks and analyzed for a variety of blood and saliva measures relevant to health promotion.

**Results:**

Curcumin, but not placebo, produced the following statistically significant changes: lowering of plasma triglyceride values, lowering of salivary amylase levels, raising of salivary radical scavenging capacities, raising of plasma catalase activities, lowering of plasma beta amyloid protein concentrations, lowering of plasma sICAM readings, increased plasma myeloperoxidase without increased c-reactive protein levels, increased plasma nitric oxide, and decreased plasma alanine amino transferase activities.

**Conclusion:**

Collectively, these results demonstrate that a low dose of a curcumin-lipid preparation can produce a variety of potentially health promoting effects in healthy middle aged people.

## Background

Curcumin extracted from the spice turmeric shows many different actions in cell cultures and experimental animals [1-3]. However, the applicability of this work to human health has been questioned due to the low absorption of curcumin from extract supplement products [1,4-6]. Even so, some studies of high doses of curcumin preparations have had some effects in people with established health problems rev in [1,2]. These doses are called high because the 1 g or more quantities used in most studies exceed what can be typically obtained by people using turmeric related spices. At least one study has tried a much lower dose of curcumin, but this study uses whole turmeric products that cannot really be called a concentrated curcumin extract ie [7]. A few studies have also tried 500 mg curcumin/day as concentrated extract ie [8,9]. Yet, this dose is still fairly high and has not always been given without another active agent ie [9]. In the latter case, the effects of the curcumin versus those of the other agent cannot easily be distinguished. One exception to this high dose approach comes from a recent study that used 180 mg/day of curcumin, but the curcumin by itself did not affect the measures under consideration [10]. In contrast to these studies, the present study examined a fairly concentrated extract at 80 mg curcumin/day, which is a much lower dose than has generally been tried in previous work. Also in contrast to most previous work, the present study used a lipid-curcumin mixture that was projected to be relatively well absorbed.

Another difference between the present study and previous human work on curcumin is that the present study examined healthy subjects rather than people with a health problem. Nearly all of the previous human studies on curcumin extracts have studied people with established health problems. One exception is a study on 500 mg curcumin/day in relatively healthy people, but the intervention lasted just 1 week [8]. More studies of healthy people are needed because the curcumin effects seen in cell cultures and experimental animals could reduce the risk of diseases not yet present.

In studies on cell cultures and experimental animals, curcumin has shown a wide range of effects. For example, curcumin has shown actions that affect lipid metabolism [11-13] various anti-inflammatory pathways [3], antioxidant reactions [1-3,14], endogenous antioxidant levels [15-18], endogenous pro-oxidant molecule concentrations[3], neurological processes[19-21] and cardiovascular physiology [1,3]. Therefore, the present study covered a wide range of potential mechanisms of action for curcumin and a wide range of health implications.

## Methods

### Subjects

The protocol was approved by The Ohio State University Human Subjects Biomedical Institutional Review Board. Apparently healthy adult males and post-menopausal females, 40–60 years old, were recruited from the Columbus, Ohio area. Potential subjects were excluded for: current major health problems, cigarette smoking, previous cardiovascular incidents, history of cancer other than small sections of skin, regular use of turmeric, dentist confirmed gingivitis, and use of supplemental phytochemical concentrates.

### Research design

Accepted subjects were assigned to either starch placebo or curcumin (N = 19/group, female/male split of 17/2, mean age ± SEM of 48 ± 6 years for the placebo and 47 ± 5 years for the curcumin). The curcumin was Longvida® Optimized Curcumin from *Curcuma Longa* root given at 400 mg powder per day containing 80 mg curcumin with each of the following ingredients as a proprietary blend: vegetable-derived stearic acid dextrin, hydroxypropylmethylcellulose (vegetarian capsule), soy lecithin, ascorbyl palmitate and silicon dioxide. Longvida® is a trademark of Verdure Sciences, Noblesville, IN, USA. Subjects consumed the assigned product for 4 weeks, with blood samples taken before and after the supplementation period. Subjects were instructed to maintain their previous dietary and exercise practices during participation.

### Laboratory analysis

After a fast of about 8 h or more, blood was collected into a tube with heparin, centrifuged at 3000 x g for 30 min to obtain plasma and erythrocytes, and the erythrocytes were washed with phosphate buffered saline, and then extracted with ethanol: chloroform as described earlier [22]. Plasma was stored at −70°C and the erythrocyte extract was stored at −20°C. Saliva was obtained without acute stimulation and stored at −70°C until assayed. Before assay, the saliva samples were briefly centrifuged in an Eppendorf Microfuge to remove solid material and precipitates.

Plasma total cholesterol, triglyceride, LDL, HDL and alanine aminotransferase (ALT) were measured using the Roche Cobas C111 Clinical Chemistry Analyzer (Indianapolis, Indiana, USA). Salivary amylase was determined using an ELISA kit from Salimetrics (State College, Pennsylvania, USA). Plasma catalase and nitric oxide were assayed using kits From Cayman Chemical Company (Ann Arbor, Michigan, USA). Plasma β amyloid protein (1–40) was measured with a commercial ELISA kit from Wako Pure Chemical Industries (Osaka, Japan). Plasma soluble intercellular adhesion molecule (sICAM) was assayed by an ELISA kit from Invitrogen (Frederick, Maryland, USA). Salivary antioxidant status was assessed as free radical scavenging capacity by a kit from Oxford Biomedical Research (Oxford, Michigan, USA). Plasma c-reactive protein was determined by a high sensitivity ELISA kit from MP Biomedicals (Solon, Ohio, USA). Plasma myeloperoxidase was measured by an ELISA kit from Assay Designs (Ann Arbor, Michigan, USA). Plasma ceruloplasmin activity was determined colorimetrically by oxidation of p-phenylenediamine as described earlier [22]. Erythrocyte superoxide dismutase was assayed by a spectrophotometric kinetic assay described before [22].

## Results

**Figure 1:**
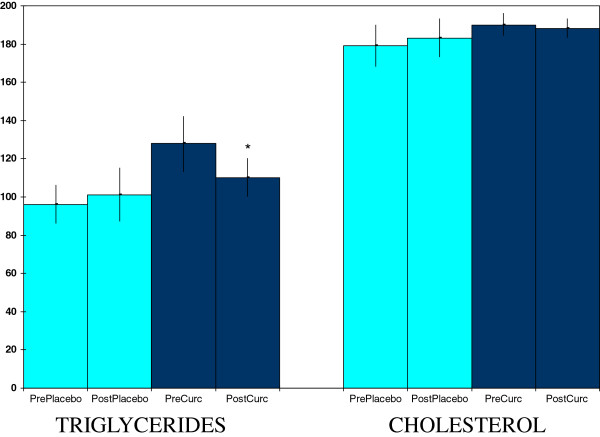
**Curcumin effects on plasma triglycerides and cholesterol concentrations (mg/dl).** Values are means ± SEM for N = 19 pre- and post-treatment of 4 weeks. *Significantly different from pre-value, paired t-test, p < 0.05.

Curcumin supplementation produced a variety of effects in plasma and saliva measures that are relevant to health promotion. Supplementation, but not placebo, lowered plasma triglyceride values (Figure 1). The curcumin effect did not extend to plasma lipids in general since no significant effects were seen for plasma total cholesterol (Figure 1) nor for LDL or HDL cholesterol (data not shown).

**Figure 2:**
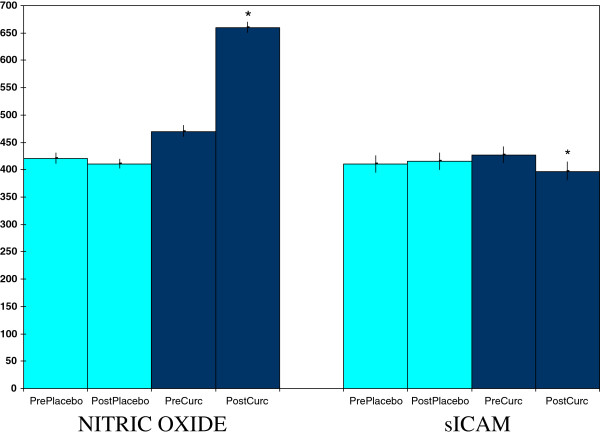
**Curcumin effects on plasma concentrations of nitric oxide (μM x 10) and soluble intercellular adhesion molecule (sICAM)(ng/ml).** Values are means ± SEM for N = 19 pre- and post-treatment of 4 weeks. *Significantly different from pre-value, paired t-test, p < 0.05.

Curcumin also affected two non-lipid related measures relevant to cardiovascular health (Figure 2). One of these effects was an increase in plasma contents of nitric oxide, a molecule that can work against high blood pressure [23]. The other cardiovascular-relevant effect was a lowering of plasma concentrations of sICAM, a molecule linked to atherosclerosis [24].

**Figure 3:**
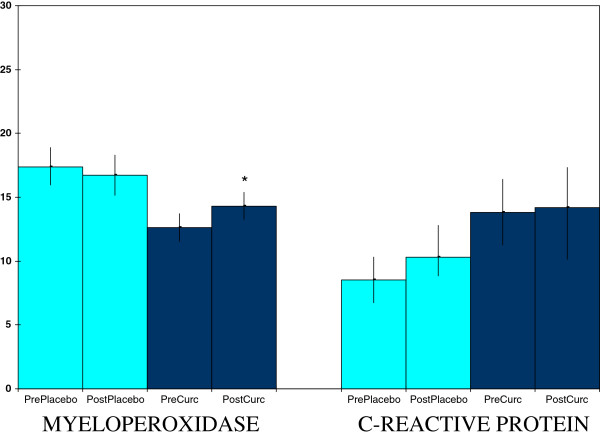
**Curcumin effects on plasma concentrations of myeloperoxidase (ng/ml) and c-reactive protein (mg/L x 10).** Values are means ± SEM for N = 19 pre- and post-treatment of 4 weeks. *Significantly different from pre-value, paired t-test, p < 0.05.

**Figure 4:**
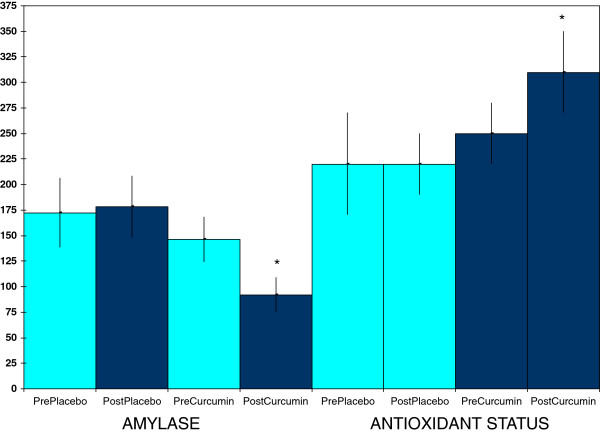
**Curcumin effects on saliva activities of amylase (U/L) and antioxidant status (μM of copper reducing equivalents).** Values are means ± SEM for N = 19 pre- and post-treatment of 4 weeks. *Significantly different from pre-value, paired t-test, p < 0.05.

**Figure 5:**
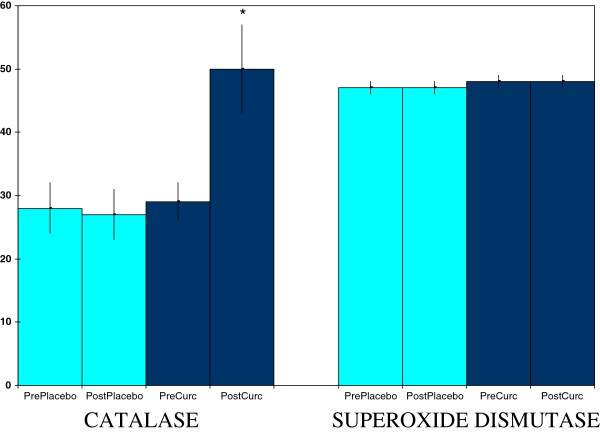
**Curcumin effects on plasma activities of catalase (U/ml) and erythrocyte superoxide dismutase (U/ml packed cells x 10^-2^).** Values are means ± SEM for N = 19 pre- and post-treatment of 4 weeks. *Significantly different from pre-value, paired t-test, p < 0.01.

Curcumin supplementation, but not placebo, raised plasma myeloperoxidase concentrations (Figure 3), a part of both normal and inflammation-related neutrophil function [25,26]. This effect was not accompanied by a rise in plasma levels of c-reactive protein (Figure 3), nor by a rise in ceruloplasmin values (data not shown), both of which can be markers of inflammation [1,22,27]. In addition, curcumin supplementation, but not placebo, lowered salivary amylase activities (Figure 4), which can mark sympathetic nervous system stress [28]. Curcumin also raised salivary radical scavenging capacities (Figure 4). The latter effect, a boosting of one determinant of antioxidant protection, was complimented by raising activities of the plasma antioxidant enzyme catalase (Figure 5). However, this last effect did not extend to all antioxidant enzymes as no effect was seen for erythrocyte superoxide dismutase activities (Figure 5) nor for plasma glutathione peroxidase (data not shown).

**Figure 6:**
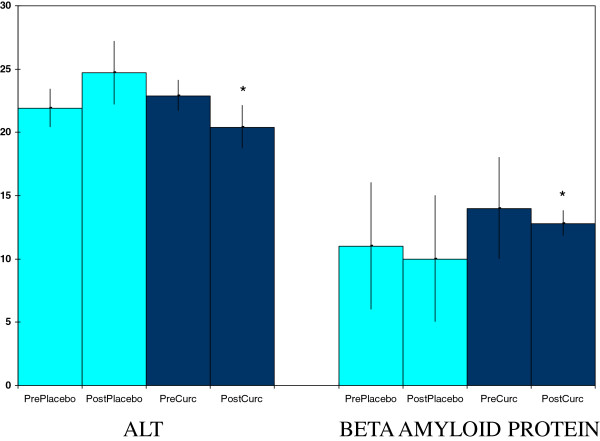
**Curcumin effects on plasma activities of alanine aminotransferase (ALT) (U/L) and beta amyloid protein (pmoles/L).** Values are means ± SEM for N = 19 pre- and post-treatment of 4 weeks. *Significantly different from pre-value, paired t-test, p < 0.05.

Curcumin supplementation, but not placebo, reduced plasma contents of beta amyloid protein (Figure 6), a maker of brain aging, especially in relation to Alzheimer’s disease [29]. Curcumin also reduced plasma alanine amino transferase activities (Figure 6), a liver injury marker [30].

## Discussion

This study demonstrated that in apparently healthy individuals, a relatively low dose of a specific curcumin preparation can exert a variety of health promoting effects. Previous human intervention studies had generally emphasized much larger doses in people with existing health problems rev in [1,2,6]. Since the relatively low dose used here did produce various bio-actions, presumably a good amount of curcumin was absorbed. However, this was not tested directly. The variety of actions seen here were tested for only one fairly narrow age range, but future studies can examine other ages.

Certain dietary bioactive compounds only show very distinct effects when counteracting a physiological stress. This is likely why curcumin has been tested largely in the presence of health problems in people and in animal models for human disease. However, the wide variety of potentially health promoting effects seen in the present study suggests that curcumin can produce benefits in people without immediate disease states. This diversity of effects seen here conforms to the diverse range of mechanisms that can be affected by curcumin in experimental animals and cell cultures [1-3]. For example, curcumin affected readings for both nitric oxide and sICAM. Both of these molecules hold relevance for cardiovascular disease risk, but from completely different perspectives. Nitric oxide relates to blood pressure [23] while sICAM relates to atherosclerosis [24]. The current nitric oxide results may seem to contradict two previous studies where curcumin reduces nitric oxide [31,32]. However, the previous studies examined inflammatory states where nitric oxide levels can be raised by an inducible synthesis enzyme. The curcumin effect seems to result from actions on this enzyme [31], which would not show much activity in the healthy, non-stressed subjects studied here.

Another effect of curcumin was reduction of plasma ALT activities, which are generally used to mark liver injury [30]. This result for curcumin is consistent with work in experimental animals with various chemically-induced liver injuries ie [3,33]. In these animals, a rise in ALT activities can be limited by administering curcumin. However, two major differences exist between those studies and the present work. First, the present study utilized a much lower curcumin dose than in the animal work, and second, the current study did not include any overt chemical injury. Thus, the animal work raises the possibility of using high dose curcumin for drug type effects, but the current work raises the possibility of using low dose curcumin for liver health maintenance. This contrasts some suggestion that high dose curcumin doses can produce liver toxicity [34].

In the present study, curcumin effects on blood lipids gave mixed results compared to experimental animal studies. Specifically, in the present study, curcumin lowered triglyceride readings but did not affect various types of cholesterol readings. In experimental animals, curcumin can affect all these readings ie [11-14]. However, it should be noted that these experimental animal models induce high cholesterol and triglyceride levels via oral intake of agents such as cholesterol, fat or alcohol. Conversely, in the present study, neither plasma cholesterol nor triglycerides were elevated by any extreme dietary intervention. Curcumin may regulate cholesterol through mechanisms that only become major effectors under certain stress conditions. In opposition to this proposition, one study finds cholesterol lowering by 500 mg curcumin/day in relatively healthy people. However, this study lasted just 1 week [8]. In one other study [35], curcumin failed to lower either cholesterol or triglyceride readings. The exact dose given cannot be ascertained since the percent curcumin of the extracts were not analyzed. The study did provide evidence that some curcumin was absorbed, but the amount absorbed may not have been enough to lower triglyceride readings.

Curcumin raised plasma myeloperoxidase, an effect often associated with neutrophil mediated inflammation [26]. Yet, in the present study, no change was seen for either c-reactive protein or ceruloplasmin concentrations, both of which rise with inflammation [22,27]. Possibly, the curcumin effect on myeloperoxidase indicated strengthened cellular immune function, not an inflammatory reaction. Curcumin is known to strengthen some aspects of cellular immunity even though it also suppresses pro-inflammatory aspects of immune function [rev in [36].

Research in experimental animals and carried out *in vitro* have raised the possibility that curcumin could work against development of Alzheimer’s disease rev in [21]. In the present study, curcumin decreased plasma beta amyloid protein concentrations, which relates to one mechanism by which curcumin may impact Alzheimer’s disease development. Although the percent decrease was not big, the decrease could become larger with a longer intervention.

In this study, curcumin showed signs of both direct and indirect antioxidant actions. The curcumin-induced increase in salivary radical scavenging capacity is consistent with a direct antioxidant action (elimination of free radicals by curcumin and/or its metabolites). Curcumin has shown this type of activity *in vitro*[2,37]. In the present study, curcumin also showed indirect antioxidant action by elevating plasma activities of the endogenous antioxidant enzyme catalase. Interestingly, low plasma catalase is associated with a high risk for one form of cardiovascular disease [38]. Experimental animal studies provide precedent for curcumin stimulation of increases in antioxidant enzyme activities [14-17]. In the present study, plasma catalase activities increased after curcumin treatment, but readings for two other antioxidant enzymes, plasma glutathione peroxidase and erythrocyte superoxide dismutase, did not change. In contrast, curcumin is reported to increase all three enzymes in humans exposed to arsenic [39]. However, this increase is not from normal activities to above normal, which is what was observed for catalase activities in the present study. In the people with arsenic exposure, low activities are partially returned to normal. Thus, the work with arsenic exposed subjects pertains to protection against toxin-induced reduction in activity, but the present study considered activity elevations above normal for three antioxidant enzymes. Although only the catalase activity showed such elevation in blood samples, activities of the other two antioxidant enzymes may have increased in other body sites.

## Conclusions

In summary, a low dose of a lipidated curcumin product produced a range of potentially health promoting actions in healthy middle aged people.

## Competing interests

The authors declare that they have no competing interests.

## Authors' contributions

RAD secured the funding, oversaw the study, and had the main writing responsibility. EJ had the main responsibility for the laboratory analysis including adapting methods to this study’s need. EJ also made some decisions on the assay methodology to be used, formatted the paper, and gave input on the study design and manuscript writing. SZ did some of the laboratory work and made decisions on some of the assay methodology to be used. SZ also gave input on the study design and manuscript writing. JB was the lead investigator for human subject research regulation compliance and contributed to study design and manuscript writing. All authors read and approved the final manuscript.
